# Heart rate changes in partial seizures: analysis of influencing factors among refractory patients

**DOI:** 10.1186/1471-2377-14-135

**Published:** 2014-06-20

**Authors:** Wei Chen, Chang-Li Guo, Pei-Song Zhang, Chong Liu, Hui Qiao, Jian-Guo Zhang, Fan-Gang Meng

**Affiliations:** 1 Department of Neurosurgery, Liaocheng People’s Hospital, Shangdong province, Liaocheng 252000, China; 2 Beijing Neurosurgical Institute, Capital Medical University, Beijing 100050, China; 3 Department of Neurosurgery, Beijing Tiantan Hospital, Capital Medical University, Beijing 100050, China

**Keywords:** Partial seizures, HR changes, Video-EEG-ECG, Influence factors

## Abstract

**Background:**

We analyzed the frequency of heart rate (HR) changes related to seizures, and we sought to identify the influencing factors of these changes during partial seizures, to summarize the regularity of the HR changes and gain some insight into the mechanisms involved in the neuronal regulation of cardiovascular function. To date, detailed information on influencing factors of HR changes related to seizures by multiple linear regression analysis remains scarce.

**Methods:**

Using video-electroencephalograph (EEG)-electrocardiograph (ECG) recordings, we retrospectively assessed the changes in the HR of 81 patients during a total of 181 seizures, including 27 simple partial seizures (SPS), 110 complex partial seizures (CPS) and 44 complex partial seizures secondarily generalized (CPS-G). The epileptogenic focus and the seizure type, age, gender, and sleep/wakefulness state of each patient were evaluated during and after the seizure onset. The HR changes were evaluated in the stage of epilepsy as time varies.

**Results:**

Of the 181 seizures from 81 patients with ictal ECGs, 152 seizures (83.98%) from 74 patients were accompanied by ictal tachycardia (IT). And only 1 patient was accompanied by ictal bradycardia (IB). A patient has both IT and IB. We observed that HR difference was independently correlated with side, type and sleep/wakefulness state. In this analysis, the HR changes were related to the side, gender, seizure type, and sleep/wakefulness state. Right focus, male, sleep, and CPS-G showed more significant increases than that were observed in left, female, wakefulness, SPS and CPS. HR increases rapidly within 10 seconds before seizure onset and ictus, and typically slows to normal with seizure offset.

**Conclusion:**

CPS-G, sleep and right focus led to higher ictal HR. The HR in the stage of epilepsy has regularly been observed to change to become time-varying. The risk factors of ictal HR need to be controlled along with sleep, CPS-G and right focus. Our study first explains that the HR in seizures has a regular evolution varying with time. Our study might help to further clarify the basic mechanisms of interactions between heart and brain, making seizure detection and closed-loop systems a possible therapeutic alternative in refractory patients.

## Background

Sudden unexpected death in epilepsy (SUDEP) represents an interesting conundrum of the heart-brain interface that is the focus of intense research. The exact pathophysiologic processes were unknown. Autonomic changes affecting the cardiac rhythm may play a role, although the precise pathways involved are still unclear [1]. The cardiac effects of epilepsy are widespread and range from subtle changes in heart rate variability (HRV) to ictal sinus arrest.

HRV reflects the beat-to-beat alterations in the HR and is mainly modulated by parasympathetic and sympathetic activity [2]. HRV can be used as a tool to show information on the functional state of the autonomic nervous system. And HRV is a mirror of neuronal influences on the cardiac pacemaker as one of the important functions of the autonomic nervous system [3]. It was found to be lower with refractory epilepsy, possibly resulting from parasympathetic or vagal reduction. This can make patients more susceptible to tachycardia and fibrillation and possibly SUDEP [4,5]. Although IB in HR during epileptic seizures can occur [6], the most common abnormality reported is Ictal tachycardia (IT) [7-9], which has been reported following CPS and SPS [4]. IT has been reported in up to 100% of seizures and can precede, coincide with, or follow ictal discharges. Analysis shows that HR increases significantly in CPS-G compared to non-generalized seizures [10]. More insight into the mechanisms of generalization of partial seizures may improve treatment, and possibly also contribute to a reduced number of SUDEP, which has been reported previously [11]. HR increase seems more prominent in temporal lobe seizures (where insular spread is common), compared with extratemporal seizures [12-14]. Whether there is a central hemispheric lateralization of cardiac autonomic control is debatable. A predominance of increase of HR as compared to baseline in right sided temporal seizures can be expected and is confirmed by some [15,16], but others describe a more prominent HR change during left-sided seizures [10]. To clarify these questions, we analyzed the frequency of cardiac rhythm related to seizures and attempted to determine risk factors and associated clinical characteristics.

Apart from SUDEP, mortality and morbidity as a result of seizure-related events (e.g., accidents and drowning) are frequent. As the occurrence of seizures is unpredictable, much research was devoted into prediction or early detection of seizures. Detection of seizures could be very helpful not only in the development of warning systems but also in novel treatment strategies [17].

## Methods

### Subjects

We analyzed HR changes on 181 consecutive partial seizures from 81 epileptic patients referred to the Department of Neurosurgery of Liaocheng People’s Hospital, Beijing Tiantan Hospital, and Beijing Neurosurgical Institute at Capital Medical University for presurgical evaluation and scheduled as candidates for surgery. Between January 2011 and June 2013, most of these patients were admitted to our unit to determine their suitability for epilepsy surgery. None of the subjects suffered from cardiovascular disease. Status epileptics have shorter interval time between two seizures, the time period from 60 seconds before EEG seizure onset to 60 seconds after seizure offset is not easy to define. Therefore, status epileptics were excluded from the analysis. For three groups of seizures (SPS, CPS and CPS-G), consecutive events providing satisfactory video – electroencephalograph – electrocardiograph (EEG-ECG) recording were analyzed. The patients with at least one qualifying event were entered consecutively. The number of seizures analyzed per patient ranged from 1 to 4 (average 2.2). The study conformed to the Declaration of Helsinki, was approved by the ethics committee of the Beijing Tiantan Hospital, Capital Medical University.

### Data

All the available inpatient video-EEG-ECG data for each subject were reviewed. Digital EEG was recorded with a common reference at a sampling frequency of 256 Hz. The EEG data were obtained by using the international standard 10–20 system electrode placement and sphenoidal electrodes with one lead of ECG monitoring. ECG was recorded (sampling frequency 256 Hz, low-frequency filter 0.3 Hz, high-frequency filter 70 Hz). The non-lateralized patients were recorded with indwelling depth electrodes. HR was assessed by manual analysis. HR were the beats per minute at different time epoch in each seizure out of EEG-ECG which was recorded within 10 seconds in each screen, then count the number of QRS complexs within 10 seconds, multiply the result by 6. The baseline ECG rates were determined for 10 second epochs and expressed as beats per minute, and we measured the HR for the 60 seconds that preceded the seizure onset as the baseline ECG. The maximum and minimum HR during seizures were similarly identified [18]. HR was calculated at the 60S, 40S, 30S, 20S, 10S before seizure onset, ictal onset, ictal, and 60S after seizure offset. Seizure onset was defined as EEG seizure onset. The first 10s of the ictus (herein called ‘ictal onset’), seizure offset was defined as the end of EEG seizure end. In CPS-G seizures, ECG signals were commonly obscured by muscle and movement artifacts. We found that near the end of the tonic-clonic phase, ECG signals were less susceptible to artifacts interference. Additionally, we can get data by reducing the sensitivity of scalp electrodes, increasing the sensitivity and low-frequency filters of ECG, and debasing high -frequency filters of ECG. Therefore, ictal HR was possible to obtain. The video-EEG data evaluated for each recorded seizure are (a) the location of the ictal onset zone, (b) the seizure duration, (c) the seizure type, and (d) the sleep/wakefulness state during which the seizure arose. All the ECG data were reviewed by a board-certified cardiologist and electrophysiologist. Magnetic resonance imaging (MRI) of the brain for each patient was obtained from a review of the medical records.

### Type (IT and IB) of HR changes and curve graph

IT and IB were defined as development of a heart rate more than 100 and less than 60 beats/min respectively after onset of seizure activity on the EEG. We measured the HR for the 10, 20, 30, 40, 60 seconds before the seizure onset, ictal onset, ictal and 60 seconds after seizure offset. We measured the HR for the 60 seconds that preceded the seizure onset as the baseline HR. The average of the difference between the HR at different time and the baseline HR can tell us whether it produce ictal HR changes. The HR changes were evaluated in the stage of epilepsy as time varies. Curve graphs of the seizures could be drawn based on the HR changes and HR.

### Statistical analysis

SPSS for Windows version 11.0 (SPSS, Chicago, IL, USA) was used for the statistical analysis. A mean baseline HR (B-HR) and maximum and minimum HR were estimated during seizures. All reported variables were assessed with a non-parametric variable test (One-Sample Kolmogorov–Smirnov), assessing if it was distributed normally. The difference between gender, hemispheric lateralization and sleep/wakefulness on the HR changes, and the difference between ictal and postictal HR during sleep and CPS-G sleep separately were assessed with an independent two–sample t-test. Analysis of variance (ANOVA) was used in comparing the mean HR difference. Post hoc correction for multiple testing has been performed. Spearman’s rank and Pearson's correlation coefficient were used. A multivariate analysis was performed by means multiple linear regressions to assess the influence of the indicators on the HR difference. The process of variable selection was stepwise forward, at the level of 5%, which selected the smallest subgroup of the independent variables that influenced the HR difference. P < 0.05 was considered statistically significant.

## Results

### Patients and MRI data

Eighty-one patients (56 male and 25 female, with a mean age of 22.8 years, range: 3–49 years) were included in this study. The mean duration of all the seizures was 152 s (the standard deviation was 57 s; the range was 3–480 s). All the patients had consistent HR changes from seizure to seizure. Brain MRIs detected morphologic alterations in 65 of 81 cases (partial epilepsy), and in 39 of 44 cases (temporal lobe epilepsy) focal lesions were documented, including hippocampal sclerosis (n = 30), cavernous hemangioma (n = 7) and arachnoid cyst (n = 2). In the morphologic alterations in 15 of 23 cases (frontal lobe epilepsy), 15 cases with focal lesions were abnormal signals, and the alterations in 11 of 14 cases (occipital lobe epilepsy), 11 cases with abnormal signals (n = 11).

### Seizure types and location of ictal onset

A total of 181 seizures from 81 patients indicated symptomatic or cryptogenic partial epilepsy, 27 had SPS seizures (14.9%), 110 had CPS but no GTC seizures (60.8%) and 44 had CPS-G seizures (24.3%). A total of 47 seizures were frontal lobe, 101 seizures were temporal lobe and 33 seizures were occipital lobe in seizure onset (Table 1).

**Table 1:** Factors influencing ictal HR

**Category**	**Number of seizures (%)**	**Maximum ictal HR mean ± S.D**	**Minimum ictal HR mean ± S.D.**	**HR difference mean ± S.D**	**p-value (HR difference)**
*Gender*					
Male	124 (68.5%)	132.3 ± 29.2	75.0 ± 13.0	57.2 ± 27.5	*P* = 0.001
Female	57 (31.5%)	123.9 ± 21.1	79.5 ± 15.1	44.5 ± 22.7	
*Age* (*years*)					
<14	32 (17.7%)	132.4 ± 24.3	89.1 ± 13.6	43.4 ± 30.9	*P* = 0.022
≥14	149 (82.3%)	129.0 ± 27.7	73.7 ± 12.2	55.3 ± 25.3	
Hemisphere of onset:					
Left	96 (53.0%)	128.4 ± 26.7	79.8 ± 14.0	48.5 ± 26.1	*P* = 0.011
Right	85 (47.0%)	131.0 ± 27.7	72.6 ± 12.5	58.5 ± 26.4	
*Localization*					
Frontal	47 (26.0%)	130.1 ± 31.4	76.8 ± 13.5	53.3 ± 28.5	(Frontal vs Temporal) *P* = 0.995
Temporal	101 (55.8%)	129.0 ± 25.2	75.7 ± 12.0	53.2 ± 24.1	(Temporal vs Occipital) *P* = 0.944
Occipital	33 (18.2%)	130.7 ± 27.1	78.1 ± 18.8	52.8 ± 31.9	(Frontal vs Occipital) *P* = 0.947
*State of consciousness*					
Asleep	72 (39.8%)	133.8 ± 27.1	70.9 ± 80.0	62.8 ± 25.4	*P* = 0.000
Awake	109 (60.2%)	126.8 ± 26.9	80.1 ± 13.4	46.8 ± 25.7	
*Type of seizure*					
SPS	27 (14.9%)	105.7 ± 22.9	72.9 ± 14.9	32.8 ± 24.4	(SPS vs CPS) *P* = 0.002
CPS	110 (60.8%)	124.8 ± 21.1	77.0 ± 14.3	47.7 ± 20.6	(SPS vs CPS-G) *P* = 0.000
CPS-G	44 (24.3%)	156.5 ± 26.7	77.2 ± 11.5	79.3 ± 22.4	(CPS vs CPS-G) *P* = 0.000

### HR changes

Of the 181 seizures from 81 patients with ictal ECGs, 152 (83.98%) seizures from 74 patients were accompanied by IT. And only 1 patient was accompanied by IB. A patient has both IT and IB. Our patients did not have an attack of asystole; however, IB was revealed in 1.10% of 181 seizures. The change in HR during a seizure was analyzed for possible influences by different factors. The HR before and during seizures was determined and correlated with the epileptogenic focus, gender, lateral hemispheric asymmetry, the types and sleep/wakefulness state. Most of generalized tonic-clonic phase onset (66%) in CPS-G seizures occurred at the 35 ~ 40s after EEG seizure onset. Generalized tonic-clonic phase characteristics in CPS-G seizures are presented in Table 2.

**Table 2:** Generalized tonic-clonic phase characteristics in CPS-G seizures

**Patient**	**Duration (s)**	**Heart rate**	
		**10S preictal**	**Ictal**
19	67	126	168
	59	114	174
21	270	114	150
	60	144	162
22	90	114	156
25	63	108	156
26	175	84	168
	145	108	168
33	70	138	168
34	65	78	180
	60	69	180
37	80	48	144
	83	72	156
38	65	102	144
44	40	108	144
45	59	126	156
	70	120	156
46	91	90	126
47	83	90	120
	73	114	114
48	87	108	168
49	97	114	144
50	65	108	174
53	115	78	120
55	83	108	162
59	73	114	126
63	50	102	156
	59	102	150
	55	102	132
65	90	168	171
	77	150	186
67	141	108	138
69	70	126	144
70	83	66	102
	68	108	126
73	72	96	174
	70	132	180
74	47	150	168
76	40	117	162
	45	114	138
77	65	84	103
	70	114	169
79	95	75	96
81	70	108	132

### HR difference

HR difference was defined as the difference of the maximum and minimum HR during seizures. Except for age and duration, all reported variables are distributed normally. The effect of influencing factors is summarized in Table 1. An effect of gender was demonstrated, and the HR changes in the male patients were higher than in the female patients (*P* < 0.01). The seizures in the patients >14 years old were accompanied by a significantly greater increase in HR than in the patients <14 years old (*P* < 0.05). Fifty-three percent of the seizures lateralized in origin to the left hemisphere, and the remaining to the right, and the HR difference in the patients with a right focus showed a significant increase over those with a left focus (*P* < 0.05). The HR difference was related to the seizure focus, and we found no significant differences among the three groups in relation to the location of the ictal onset zone.

**Figure 1:**
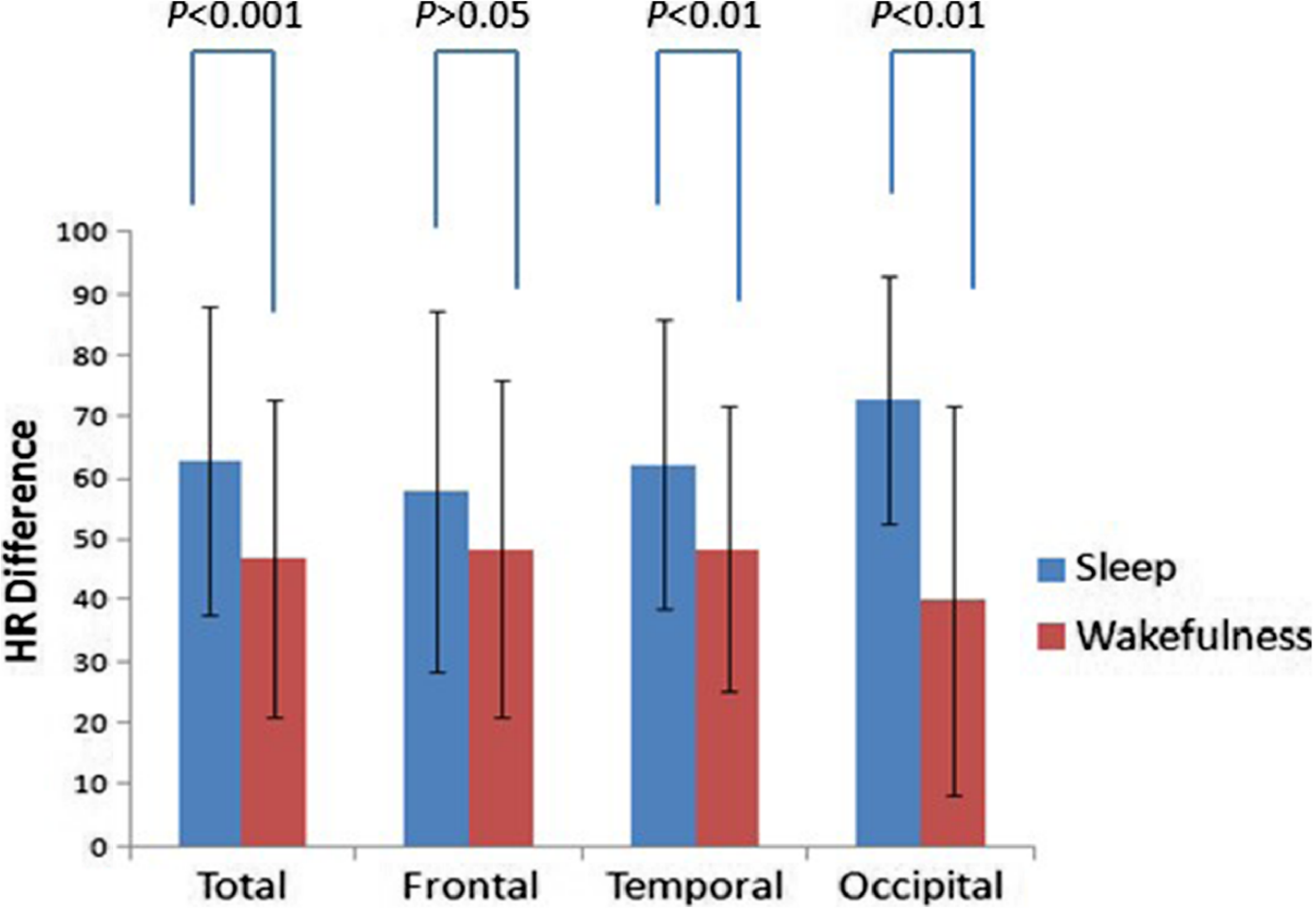
HR difference related to 181 seizure onset (Frontal: Sleep 24, Wakefulness 23; Temporal: Sleep 35, Wakefulness 66; Occipital: Sleep 13, Wakefulness 20) and sleep/wakefulness state.

**Figure 2:**
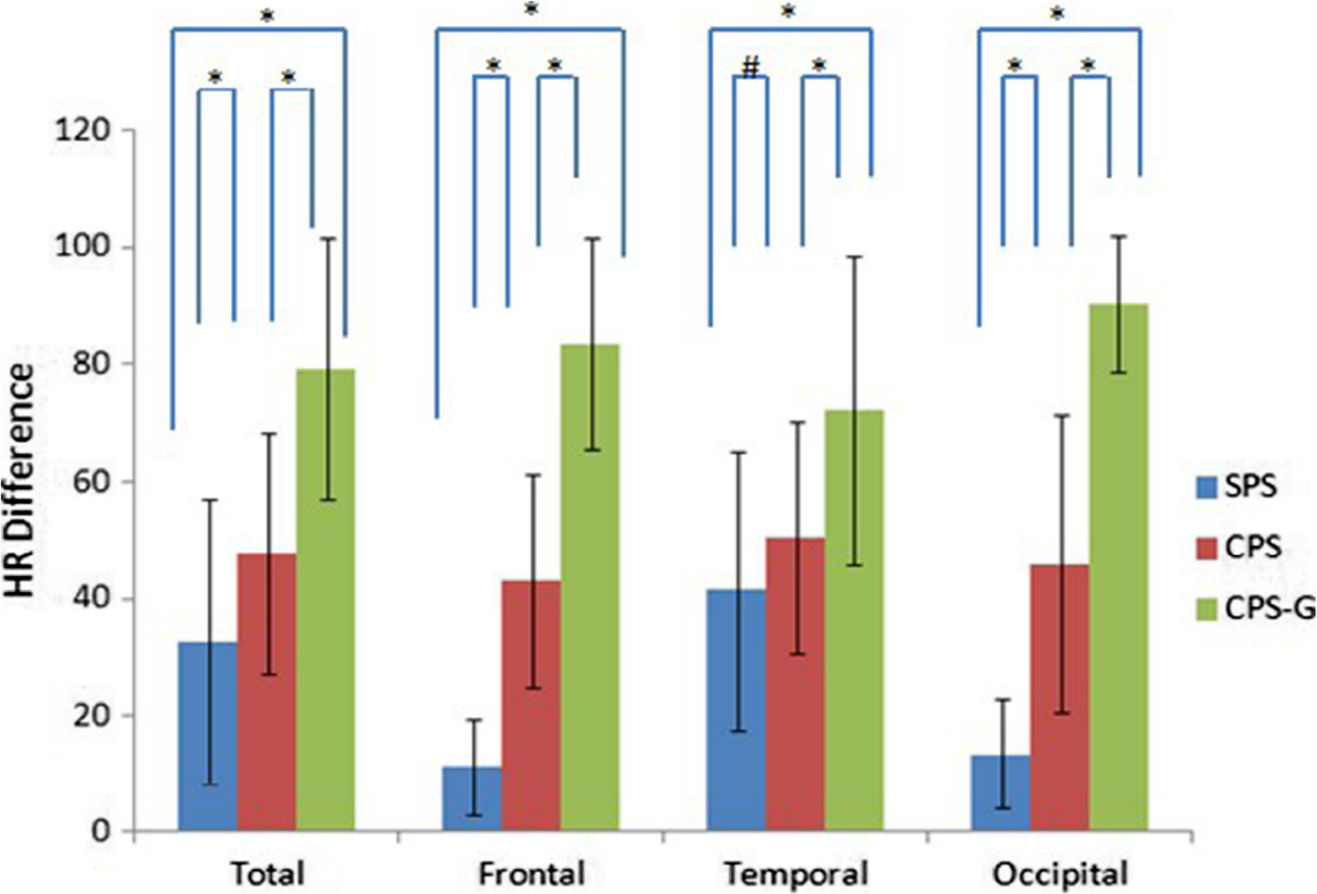
HR difference related to 181 seizure (Frontal: SPS 4, CPS 28, CPS-G 15; Temporal: SPS19, CPS 61, CPS-G 21; Occipital: SPS 4, CPS 21, CPS-G 8) onset and type (*P < 0.01, #P > 0.05).

**Figure 3:**
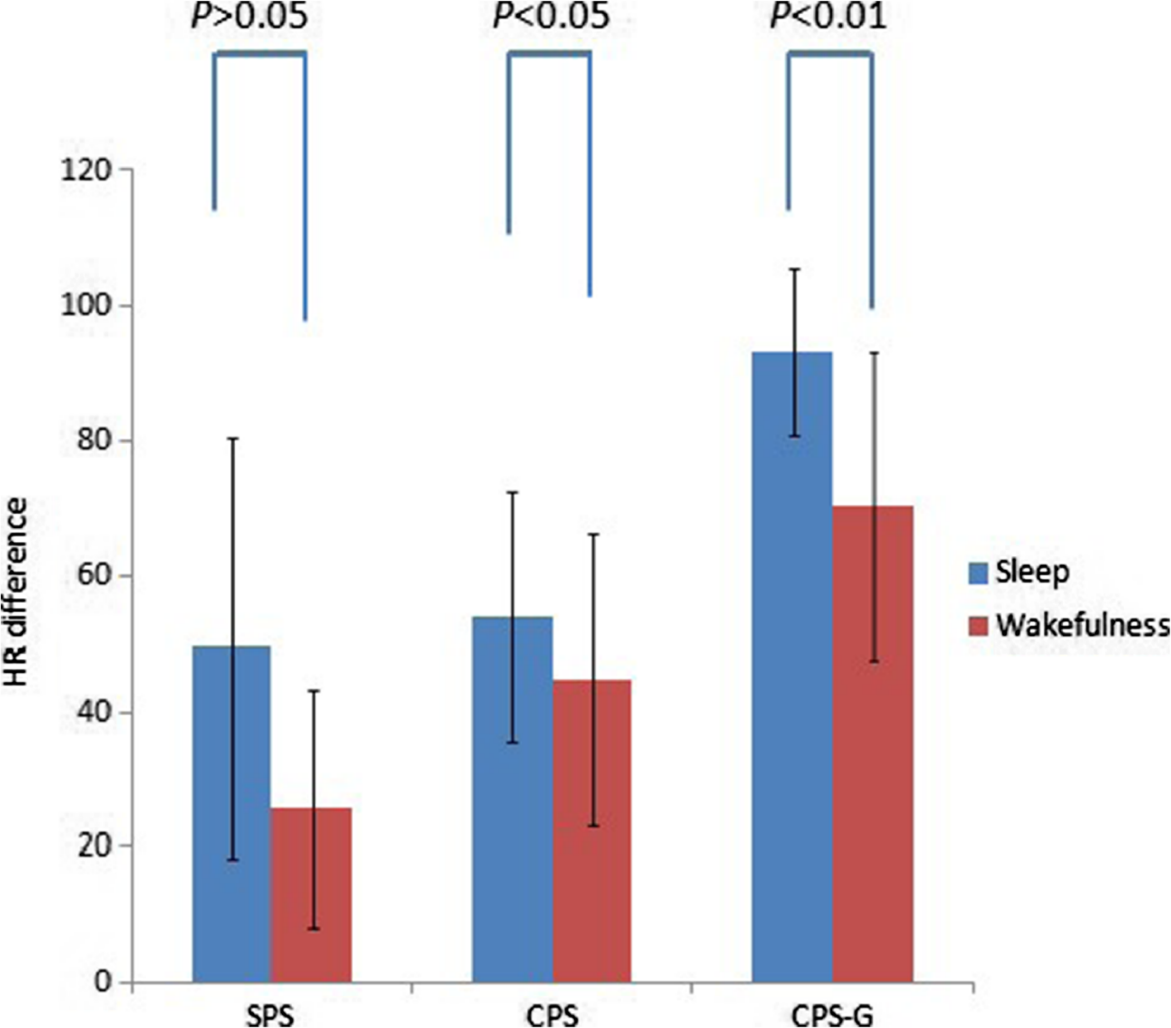
HR difference related to 181 seizure type and sleep/wakefulness state (SPS: Sleep 8, Wakefulness 19; CPS: Sleep 47, Wakefulness 63; CPS-G: Sleep 17, Wakefulness 27).

The seizure type, side and state of consciousness (sleep vs. wakefulness) showed some influences on the HR. The mean HR differences in sleep and wakefulness were (62.8 ± 25.4) and (46.8 ± 25.7) BPM, respectively. HR changes in sleep were higher than in wakefulness (*P* < 0.01) (Table 1, Figure 1). The mean HR differences of SPS, CPS and CPS-G were (32.8 ± 24.4), (47.7 ± 20.6) and (79.3 ± 22.4) BPM, respectively. With regard to the three seizure types, there were differences in the HR changes between the frontal lobe and the occipital lobe. In the SPS and CPS seizures, no differences were shown, and the remaining had an obvious statistical significance in temporal lobe seizures (Figure 2). The HR changes in the CPS-G seizures were the highest of the three types (*P* < 0.01) (Figure 2). In the CPS and CPS-G seizures (Figure 3), a greater degree of HR increase was observed during the CPS and CPS-G seizures arising from sleep than during those arising from wakefulness. This difference was observed independently with the CPS seizures (mean increase, 54.0 BPM in sleep, SD = 18.5; 44.8 BPM in wakefulness, SD = 21.8; *P* = 0.025) and tended to be observed with the CPS-G seizures (mean increase, 93.3 BPM in sleep, SD = 12.4; 70.5 BPM in wakefulness, SD = 22.9; *P* = 0.000) (Figure 3). There were difference during 72 sleep seizures and in the CPS-G sleep seizures between postictal and ictal HR. Ictal HR were higher than postictal HR in the 72 sleep seizures (P = 0.000). In the CPS-G seizures, ictal HR were higher than postictal HR. This difference was observed with the CPS-G seizures (mean, 162.6 BPM in the ictal HR, SD = 12.9; 91.8 BPM in the postictal HR, SD = 25.7; P = 0.000).

### Relation of the HR difference and various variables

A total of 152 seizures associated with IT were included in the study. As shown in Table 3, the factors with a statistically significant association with an increased HR difference among the seizures were male (*P* =0.001), right side (*P* =0.010), CPS-G type of seizure (*P* = 0.000), and sleep state (P = 0.000). There was no statistically significant (*P* >0.05) relationship between the mean HR difference of the seizures and the seizure localization.

**Table 3:** Mean difference of the HR and various variables of 169 seizures

**Variables**	**N**	**HR difference (mean)**	**F value**	**P value**
***Gender***				
Male	107	63.432	0.681^#^	0.001*
Female	45	48.933		
***Localization***				
Frontal	36	63.383	0.874^#^	0.419
Temporal	89	57.081		
Occipital	27	60.267		
***Side***				
Left	80	54.270	0.100^#^	0.010*
Right	72	64.550		
***Type***				
SPS	14	47.486	32.050^#^	0.000*
CPS	95	51.196		
CPS-G	43	80.484		
***State***				
Sleep	64	67.388	0.297^#^	0.000*
Wakefulness	88	53.141		

^#^Value between groups. *Statistically significant.

Regarding the influence of the course of illness, no statistically significant association was observed (*P* >0.05). Table 4 presents the correlation between the HR difference and the clinical variables. We observed that the HR difference presented moderate correlations with duration (*P* = 0.000). There was no statistically significant (*P* > 0.05) correlation between the mean HR difference of the seizures and age. We found that the HR difference presented moderate correlations with side, type, and sleep/wakefulness state (Table 5). We also found that side, type, and sleep/wakefulness state were independently predictive of the HR differences (Table 6).

**Table 4:** Correlations between the HR difference and the clinical variables

**Variables**	**Difference of heart rate**	
	**r**_**s**_	**p-value**
Age	0.018	0.824
Duration	0.307	0.000*

Spearman’s rank correlation coefficient or Spearman’s rho; *Correlation is significant at the 0.01 level (two-tailed test).

**Table 5:** Correlations between the HR difference and the clinical variables (multiple linear regression)

**Model**	**Coeficient**	**Standard error**	**p-value**
Gender	-2.151	3.833	0.576
Side	9.502	3.258	0.006
Type	-18.478	3.268	<0.001
State	13.041	3.257	<0.001
Duration	0.053	0.032	0.099

**Table 6:** HR difference related to different side, type and state

**Model**	**Coeficient**	**Standard error**	**p-value**
Side	9.635	3.232	0.003
Type	-21.382	2.751	0.000
State	13.150	3.266	0.000

Dependent variable: HR difference.

### Regularity of HR changes

**Figure 4:**
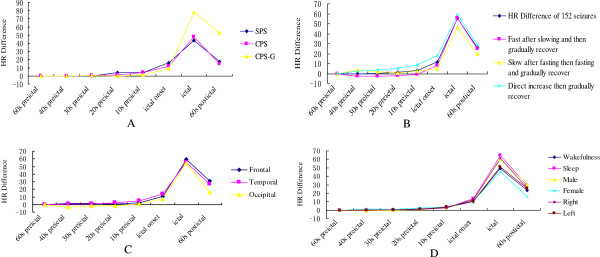
**HR changes in the regularity graph of 152 ictal tachycardia seizures. A**. HR changes in the regularity graph of three types of IT. **B**. HR changes in the regularity graph of different seizure type; **C**. HR changes in the regularity graph of different seizure onset; **D**. HR changes in the regularity graph of different seizure gender, hemispheric lateralization and state.

**Figure 5:**
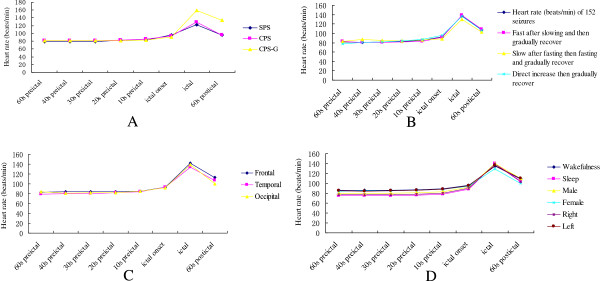
**HR in the regularity graph of 152 ictal tachycardia seizures. A**. HR in the regularity graph of three types of IT. **B**. HR in the regularity graph of different seizure type; **C**. HR in the regularity graph of different seizure onset; **D**. HR in the regularity graph of different seizure gender, hemispheric lateralization and state.

We assessed the changes of HR in 181 seizures in 81 patients. IT was found in 152 seizures (83.98%; 152/181) and IB was found in 2 seizures (1.10%; 2/181). IT was divided into three types based on the HR and HR changes at each time in each seizure (Figures 4, 5). First, the HR became fast after the slow period and then gradually recovered in 80 of 152 seizures (52.63%) as compared to baseline. Second, the HR became slow after the fast period, became fast and gradually recovered in 15 of 152 seizures (9.87%) as compared to baseline. Third, the HR increase rapidly, then gradually recovered in 57 of 152 seizures (37.50%) as compared to baseline (Figures 4, 5). No further study was conducted on IB, because the numbers were limited.

## Discussion

Addressing the issue of cardiac changes during ictal phase is challenging, the results of previous studies were different. The expected consequence of seizure activity is an increased HR as compared to baseline due to sympathetic discharge, furthermore, study showed that cardiac rhythm abnormalities occur during or after seizure activity in patients with refractory epilepsy. Seizure-related disturbances of cardiac rate, rhythm, and conduction have been identified in patients with epilepsy, with IT being the most frequent [19]. The consequence of a sustained tachycardia during epileptic seizures could be a ventricular tachycardia and sudden death. This is especially significant for individuals underlying cardiac disease [20]. These results suggest the possibility of increase SUDEP during seizure period, which is introduced by previous studies [21,22].

We noted that seizures are associated with a marked increase in HR in most cases as noted previously [19]. As shown in Table 1, there were differences in the HR changes between SPS, CPS and CPS-G (*P* < 0.01). We observed that CPS-G displayed a significantly higher ictal and postictal HR difference than SPS and CPS, which has been reported previously [23]. The observed alterations could potentially facilitate sudden cardiac death and might contribute to the association of SUDEP in epilepsy with CPS-G. There is evidence of a hemispheric-specific organization of this response as shown in the depth electrode studies by Oppenheimer et al. With the pressor response lateralized to the right and depressor response to the left hemisphere [24]. As illustrated in Table 1, the HR in the right focus showed a significant increase over that in the left focus (*P* < 0.05).

Another major finding of our study was a central imbalance of autonomic cardiac control in men only. From published reports, there is evidence for gender differences in cardiac sympathovagal regulation. HR changes in women have been shown to be significantly less than in men as a result of a reduced sympathetic influence on the heart [25]. In our study, the HR changes of 124 male seizures were higher than those of 57 female seizures (*P* < 0.001).

The relationship between the circadian clock and epilepsy might provide additional understanding of the mechanisms that induce seizures to occur at certain times [26]. As shown in Table 1, a total of 72 (39.8%) of the seizures occurred in sleep, and 109 (60.2%) occurred in wakefulness. A previous study analyzed fourteen patients who died during sleep; two patients were awake among 16 SUDEP cases. Greater increases in HR were associated with seizures arising from sleep (78 BPM increase) than from wakefulness (47 BPM; *P* < 0.001) in SUDEP cases [27]. Our study showed significant differences in HR increases which can be observed during seizures arising from sleep more frequently than during seizures arising from wakefulness (the mean increase in HR, 63 BPM in sleep and 47 BPM in wakefulness, *P* < 0.001) (Table 1). As illustrated in the Figure 3, we also find that CPS-G seizures tend to arise from sleep. However, a recent study illustrated that seizures arising from sleep which display higher HR changes are somewhat trivial, as baseline and preictal HR are lower during sleep because of higher vagal tone [28]. To minimize the bias due to sleep-related prominent vagal tone, we have compared the difference during 72 sleep and 17 CPS-G sleep seizures between postictal and ictal HR respectively. Ictal HR were higher than postictal HR in the 72 sleep seizures (P < 0.001). In the CPS-G seizures, ictal HR were higher than postictal HR (P < 0.001).

The circadian distributions of seizures arising from individual regions among seizure localizations have been poorly described in the literature. Our findings confirm that there were differences in the HR changes during wakefulness and sleep in frontal, temporal and occipital lobe seizures (*P* < 0.01) (Figure 1).

In our report, we observed that side (left/right), type (SPS, CPS and CPS-G) and state (sleep/wakefulness) were independently related to HR differences by multiple linear regression analysis (Table 6). One limitation of our regression model is the relatively few seizures after the larger dataset was divided into the various seizure types, frequently referred to as the “curse of dimensionality”. It was for this reason that we could look at sleep/wakefulness, left/right, and types of seizures together, but not add location to that analysis. Hence, we were unable to determine if a particular seizure type was more likely to occur in a specific location and whether this location differed depending on the sleep state. Our results add to the available literature by providing additional details on seizure types. Of the 181 seizures with ictal ECGs, 152 seizures (83.98%) were associated with IT. We have documented IT during both left 80 and right 72 onset seizures among 152 seizures. From Table 3, we can add location to that analysis. Whether there is a central hemispheric lateralization of cardiac autonomic control is debatable. a recent intracranial EEG-study comparing HR modulation during left-and right-sided seizures within the same patients has not found a group effect of the side of seizure-onset [29]. However, our results confirm that the HR of the patients with a right focus showed a significant increase over the HR of the patients with a left focus (*P* = 0.01), which has been reported previously [15,16]. The mean HR differences of SPS, CPS and CPS-G were (47.5 ± 24.9), (51.2 ± 19.8) and (80.5 ± 21.3) BPM, respectively. The HR changes in CPS-G seizures were the highest of the three types (*P* < 0.01) (Table 3), which has been reported previously [23,11].

The mean HR differences of sleep and wakefulness were (67.4 ± 22.9) and (53.1 ± 24.2) BPM. The HR changes during sleep are higher than in wakefulness (*P* < 0.01) (Table 3), as in previous studies [30].

Epileptic discharges of inpatients with seizures are thought to propagate to the central autonomic network and change or disturb normal autonomic control of vital cardiac functions. This activation of the central autonomic nervous system is responsible for the periictal autonomic cardiac symptoms observed in patients with epilepsy. As we know, HR changes can precede clinical and encephalographic seizure onset, and early detection of these changes can have an application in seizure detection systems [17]. Similarly, changes in vagus nerve activity—indicative of the autonomic control of the heart—could be used as seizure predictors.

So far, nobody shows the regularity of HR changes in seizures. Our study confirms that the HR in the stage of epilepsy has a regular evolution varying with time (Figures 4, 5). According to Figures 4 and 5, IT was divided into three types based on the HR at each time in each seizure. We also find that in a total of 152 investigated IT seizures, HR increases rapidly (within 10 s before seizure onset and ictus) and typically slows to normal with seizure offset (Figures 4, 5). In previous reports, ictal HR increases as compared to baseline preceded seizure onset on surface EEG for 8–19 s [7,15]. In our study population, the time lag was 10 s, conforming to the time window previously. Our curve graphs can be more intuitive to show HR changes regularity, seizure detection and help to clarify further the basic mechanisms of interactions between heart and brain. Similarly, identification of these early autonomic manifestations in seizures can theoretically contribute in developing new treatment strategies based on seizure detection for patients with refractory seizures. If further investigation validates such techniques, changes in HR might be included in seizure warning systems or even in “on demand” electrical stimulation therapies such as vagus nerve stimulation (VNS). Therefore, the monitoring of cardiac activity could predict on-coming seizures prior to their EEG onset, which could theoretically increase the quality of life and treatment efficiency in at least some patients with refractory epilepsy.

### Limitations

There are several limitations to this retrospective study, and much work remains. For future studies, it would be desirable to have more detailed information regarding certain possible risk factors for SUDEP such as EKG abnormalities, seizure etiology, IQ test performance and AED doses and serum levels.

## Conclusions

In conclusion, our results emphasize the importance of screening data on seizures for a right focus, CPS-G and sleep. This study suggests that patients with evidence of a great degree of change in autonomic tone during seizures might be at increased risk for SUDEP. Our study first explains that the HR in the stage of epilepsy has a regular evolution varying with time. Curve graphs show that HR increases rapidly within 10 seconds before seizure onset and ictus, and typically slows to normal with seizure offset. Our study might help to clarify further the basic mechanisms of interactions between heart and brain, making seizure detection and closed-loop systems a possible therapeutic alternative in refractory patient.

## Abbreviations

SUDEP: Sudden unexpected death in epilepsy; HRV: Heart rate variability; EEG–ECG: Electroencephalograph–electrocardiograph; SPS: simple partial seizures; CPS: Complex partial seizures; CPS-G: Complex partial seizures secondarily generalized; HR: Heart rate; MRI: Magnetic resonance imaging; IT: Ictal tachycardia; IB: Ictal bradycardia; BPM: Mean, beats/min; VNS: Vagus nerve stimulation.

## Competing interests

The authors declared no potential conflicts of interest with respect to the research, authorship, finance and/or publication of this article.

## Authors’ contributions

Author contributions to the study and manuscript preparation include the following. Conception and design: WC, FGM. Acquisition of data: WC, FGM, CLG, PSZ, JGZ, HQ. Analysis and interpretation of data: CL, PSZ. Drafting the article: WC, FGM. Critically revising the article: all authors. Reviewed final version of the manuscript and approved it for submission: all authors. Statistical analysis: CL, CLG, PSZ. Study supervision: FGM.

## Pre-publication history

The pre-publication history for this paper can be accessed here:

http://www.biomedcentral.com/1471-2377/14/135/prepub
